# Disease-associated myeloid states in cardiac remodeling: A new era of cardiac inflammation research enabled by computational biology

**DOI:** 10.1016/j.jphyss.2026.100107

**Published:** 2026-10-10

**Authors:** Masanori Obana, Masashi Tomimatsu, Shota Tanaka, Keisuke Shirakura, Yoshiaki Okada, Yasushi Fujio

**Affiliations:** a Laboratory of Clinical Science and Biomedicine, Graduate School of Pharmaceutical Sciences, The University of Osaka, Osaka, Japan; b Institute for Open and Transdisciplinary Research Initiatives (OTRI), The University of Osaka, Japan; c Global Center for Medical Engineering and Informatics (MEI), The University of Osaka, Japan; d Radioisotope Research Center, Institute for Radiation Sciences, The University of Osaka, Japan; e Laboratory of Cardiovascular Science and Drug Discovery, Graduate School of Pharmaceutical Sciences, The University of Osaka, Japan; f Center for Infectious Disease Education and Research, The University of Osaka (CiDER), Japan

**Keywords:** Cardiac remodeling, Myeloid cells, Single-cell transcriptomics, GARP-expressing myeloid cells

## Abstract

Adverse cardiac remodeling involves cardiomyocyte dysfunction, fibrosis, vascular alterations, and inflammation, contributing to heart failure progression. Although myeloid cells are regulators of cardiac remodeling, therapeutic strategies targeting inflammation have shown limited clinical benefit, highlighting the need for a more precise understanding of myeloid cell functions. Recent advances in single-cell transcriptomics and computational approaches have revealed substantial heterogeneity among cardiac myeloid cells and identified multiple disease-associated myeloid states with distinct molecular and functional characteristics. These findings have shifted the field beyond conventional cell classification toward understanding how specific myeloid states emerge and influence tissue remodeling through interactions with neighboring cells. In this review, we summarize recent advances in disease-associated macrophage and neutrophil states during cardiac remodeling and highlight GARP-expressing myeloid (GEM) cells as a newly identified myeloid state that promotes adverse remodeling. Understanding how disease-associated myeloid states emerge and function within multicellular tissue environments may provide new insights into cardiac remodeling.

## Highlights

• Myeloid cells, including macrophages and neutrophils, have diverse and context-dependent roles in cardiac remodeling.
• Recent advances in single-cell transcriptomics and computational approaches have revealed cardiac myeloid cell heterogeneity.
• GARP-expressing myeloid (GEM) cells are a newly identified disease-associated myeloid state that regulates cardiac remodeling.

## Introduction

Heart failure remains a major global health challenge and is a leading cause of morbidity and mortality worldwide [1], [2]. Despite significant advances in pharmacological and device-based therapies, the prognosis of patients with heart failure remains unsatisfactory [3]. Pathological cardiac remodeling, characterized by cardiomyocyte dysfunction, fibrosis, vascular alterations, and chronic inflammation, represents a central process underlying the progression of diverse cardiovascular diseases toward heart failure [4]. Traditionally, investigations of cardiac remodeling have focused primarily on cardiomyocytes and their intrinsic responses to injury or stress. However, it has become increasingly evident that the diseased heart functions as a complex multicellular environment in which interactions among multiple cell types critically influence disease progression.

Among the non-cardiomyocyte cell populations residing in the heart, immune cells have emerged as key regulators of tissue homeostasis and remodeling [4]. In particular, myeloid cells, including monocytes/macrophages and neutrophils, actively participate in both physiological adaptation and pathological responses following cardiac injury [5], [6]. These cells influence a broad range of biological processes, including inflammatory signaling, extracellular matrix remodeling, angiogenesis, and tissue repair. Importantly, these cells regulate neighboring cardiomyocytes, fibroblasts, endothelial cells, and lymphocytes through diverse intercellular signaling mechanisms, thereby coordinating local tissue responses.

Recent advances in single-cell transcriptomics, spatial transcriptomics, and other high-dimensional profiling technologies have transformed our understanding of cardiac myeloid biology [7], [8]. These approaches have revealed remarkable heterogeneity among cardiac myeloid populations and identified multiple disease-associated cellular states that emerge during myocardial infarction, pressure overload, metabolic disorders, and heart failure. Rather than functioning as uniform inflammatory effectors, myeloid cells exhibit dynamic transcriptional programs that govern tissue injury, repair, fibrosis, angiogenesis, and immune regulation in a context-dependent manner.

These discoveries have expanded the scope of cardiovascular research beyond the identification of individual cell populations toward a more comprehensive understanding of how disease-associated myeloid states shape tissue remodeling within their local cellular environment. Defining how these states emerge and influence neighboring cells during disease progression represents an important challenge in cardiovascular research.

In this review, we summarize recent advances in our understanding of disease-associated myeloid states during cardiac remodeling, focusing on their emergence, functional diversity, and contributions to pathological tissue responses (Fig. 1). We further discuss how these myeloid states interact with surrounding cells and influence cardiac remodeling, and highlight glycoprotein A repetitions predominant (GARP; LRRC32)-expressing myeloid (GEM) cells as a newly identified disease-associated myeloid state with potential roles in pathological cardiac remodeling.

**Fig. 1:**
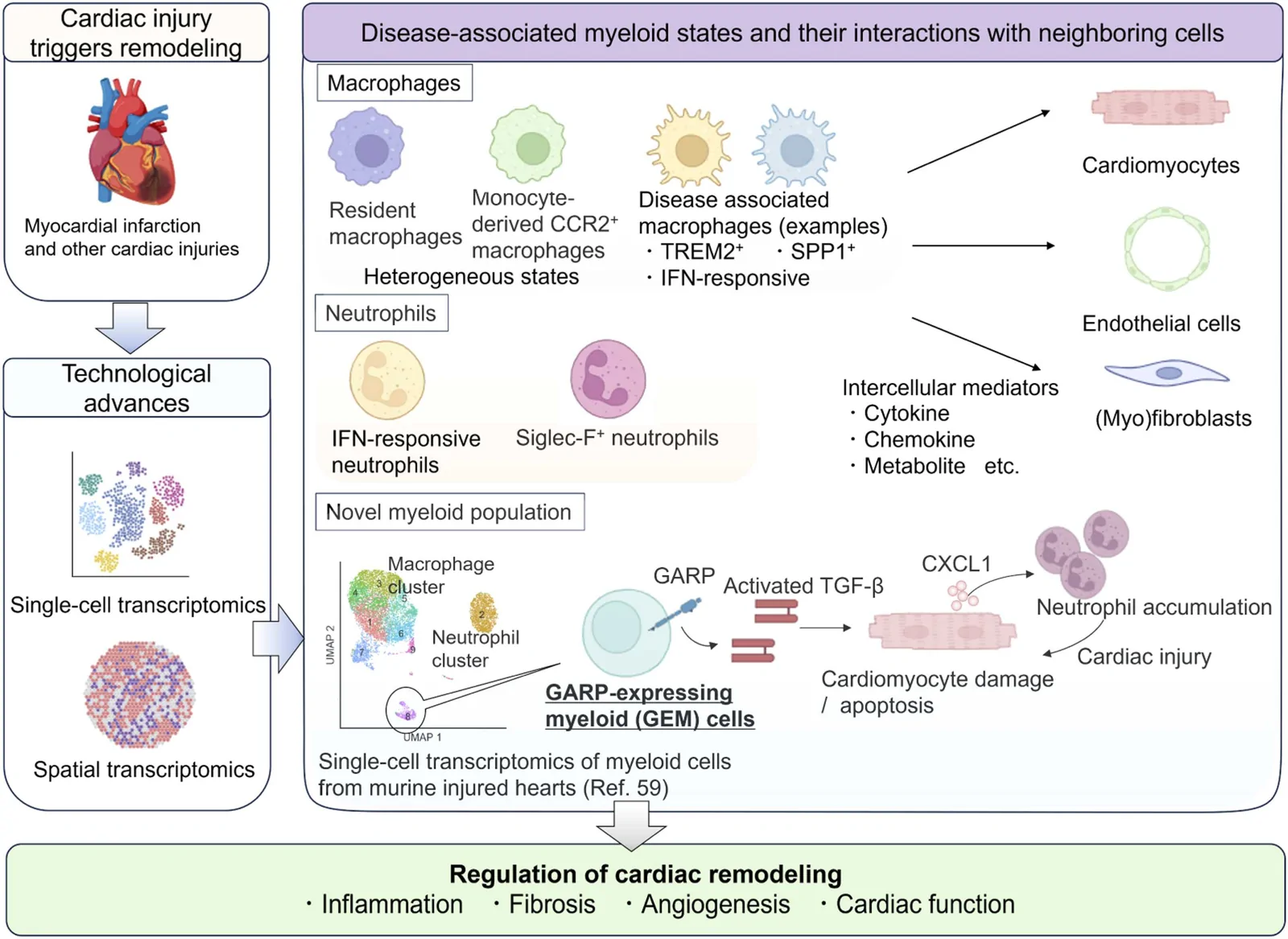
Disease-associated myeloid states and their emerging roles in cardiac remodeling.

### From cellular identity to disease-associated myeloid states: evolving perspectives on cardiac remodeling

For decades, studies of cardiac remodeling have primarily focused on the behavior and molecular functions of individual cell populations, including cardiomyocytes, fibroblasts, endothelial cells, and immune cells. These reductionist approaches have provided important mechanistic insights into cardiovascular disease; however, they are insufficient to fully explain the complexity of pathological remodeling, which arises from interactions among multiple cell types within the diseased heart.

Accumulating evidence indicates that the diseased heart functions as a highly integrated multicellular system in which diverse cell populations continuously exchange molecular signals and collectively determine tissue outcomes. Cardiomyocytes, fibroblasts, endothelial cells, and immune cells form highly interconnected communication networks through the exchange of soluble mediators, extracellular vesicles, and direct cell-cell interactions [9], [10], [11]. These dynamic interactions coordinate cellular responses within the diseased myocardium, thereby influencing inflammation, tissue remodeling, and the progression of cardiovascular disease. Consequently, pathological remodeling is increasingly recognized as a multicellular process driven by coordinated interactions among diverse cardiac cell populations rather than dysfunction of any single cell type [12].

Recent advances in single-cell and spatially resolved technologies have accelerated this conceptual transition. These approaches have revealed extensive cellular heterogeneity within the myocardium and suggested previously unrecognized relationships between distinct cellular states [13], [14], [15]. They have demonstrated that cells sharing a common lineage may participate in fundamentally different signaling programs depending on their activation state, anatomical location, and disease context. Such findings have challenged conventional classifications based solely on cell identity and have highlighted the importance of understanding functional relationships between cellular populations.

Beyond identifying previously unrecognized cell populations, recent computational approaches have provided new frameworks for predicting potential intercellular interactions from single-cell transcriptomic data. By integrating ligand-receptor interactions with gene expression profiles and spatial information, these methods provide insight into the potential directionality and cellular specificity of signaling pathways operating within the injured myocardium. Such analyses have suggested previously unappreciated signaling relationships between distinct cellular populations, providing new hypotheses regarding the cellular interactions involved in cardiac remodeling.

As a result, cardiovascular research has increasingly moved toward a multicellular perspective in which disease progression is understood as an outcome of coordinated interactions among diverse cell populations. Within this framework, remodeling is governed by dynamic signaling networks that connect cardiomyocytes, fibroblasts, endothelial cells, and immune cells. Identifying the key cellular components of these networks and defining how they coordinate tissue responses have become central objectives in contemporary cardiovascular biology. Among these cellular populations, myeloid cells represent an ideal system for understanding how disease-associated cellular states emerge and shape cardiac remodeling, owing to their remarkable heterogeneity, functional plasticity, and dynamic interactions with the tissue environment.

### From myeloid cell identity to disease-associated myeloid states in cardiac remodeling

Cardiac remodeling following injury is strongly influenced by immune responses, particularly those mediated by myeloid cells. Neutrophils are among the first immune cells recruited to the injured myocardium and play critical roles in initiating acute inflammation. Monocytes have been reported to rapidly infiltrate the infarct area after acute myocardial infarction, suggesting they may play a role in attracting neutrophils [16], [17]. Within hours after tissue injury, neutrophils accumulate at the site of damage, where they facilitate the clearance of necrotic debris through the release of inflammatory cytokines, chemokines, reactive oxygen species (ROS), and matrix metalloproteinases (MMPs). However, excessive neutrophil activation may exacerbate myocardial injury through ROS production and the formation of neutrophil extracellular traps (NETs) [18], [19], [20]. In addition to their pro-inflammatory functions, neutrophils contribute to subsequent fibrotic remodeling by promoting fibroblast activation and extracellular matrix deposition [21].

Subsequently, macrophages infiltrate the injured myocardium and participate in both inflammatory amplification and tissue repair, thereby influencing the overall remodeling process [22], [23]. Mediators released from stressed or injured cardiomyocytes activate infiltrating macrophages and shape their functional phenotypes. Traditionally, macrophages have been broadly classified into pro-inflammatory M1 and anti-inflammatory M2 phenotypes. M1 macrophages promote inflammatory responses through the production of cytokines, including tumor necrosis factor (TNF)-α, interleukin (IL)-1β, and IL-6, whereas M2 macrophages facilitate tissue repair, angiogenesis, and extracellular matrix remodeling through the secretion of anti-inflammatory and profibrotic factors, such as IL-10 and transforming growth factor (TGF)-β. This framework has provided a useful basis for understanding macrophage functions during cardiac remodeling.

Beyond their conventional classification as inflammatory or reparative cells, studies over the past decade have demonstrated that myeloid cells possess specialized molecular programs that directly influence cardiac remodeling. For example, myeloid-derived leucine-rich α2-glycoprotein (LRG) and runt-related transcription factor 2 (Runx2) have been implicated in the regulation of angiogenesis and the attenuation of adverse cardiac remodeling after myocardial infarction [24], [25]. Importantly, Runx2 expression was detected in macrophages exhibiting both M1- and M2-associated markers. These findings suggest that the biological functions of cardiac myeloid cells cannot be fully explained by broad phenotypic classifications alone.

Accordingly, cardiac myeloid cells are increasingly recognized as a heterogeneous population composed of multiple functionally specialized populations. As exemplified by Runx2-positive macrophages, which exhibited both M1- and M2-associated features, macrophage activation cannot be fully captured by the traditional M1/M2 paradigm [26], as individual populations frequently display overlapping transcriptional and functional features. Similarly, emerging evidence indicates that neutrophils also exhibit context-dependent functions beyond their classical role as short-lived inflammatory effector cells [27]. Moreover, these findings suggest that distinct myeloid states may exert specialized effects on cardiac remodeling through context-dependent interactions with neighboring cells, including cardiomyocytes, fibroblasts, endothelial cells, and other immune cells.

Recent advances in single-cell RNA sequencing and spatial transcriptomics have substantially expanded our understanding of this complexity [14], [28]. These technologies have revealed remarkable heterogeneity among cardiac myeloid cells and identified multiple transcriptionally distinct disease-associated states that emerge during cardiac injury and remodeling. Importantly, they have also provided new opportunities to examine how these states relate to the surrounding cellular environment.

### Disease-associated macrophage states and their functional roles in cardiac remodeling

Cardiac macrophages comprise a heterogeneous population that undergoes marked changes during cardiac remodeling. Studies using lineage tracing, fate mapping, and more recently single-cell transcriptomics have identified multiple macrophage populations with distinct origins and functions, including resident macrophages, monocyte-derived macrophages, and several disease-associated macrophage states [8], [29], [30], [31].

Under physiological conditions, the myocardium contains resident macrophage populations that contribute to tissue homeostasis, immune surveillance, and electrical conduction [11]. Among them, T-cell immunoglobulin and mucin domain-containing protein 4 (TIMD4)^+^lymphatic vessel endothelial hyaluronan receptor 1 (LYVE1) ^+^MHC-II^low^CCR2^-^macrophages represent a major resident population that is largely maintained through local self-renewal and exhibits transcriptional profiles distinct from infiltrating monocyte-derived macrophages [32]. Following myocardial infarction, resident macrophages are rapidly depleted from the infarcted myocardium, and their selective ablation using a diphtheria toxin receptor-mediated depletion system exacerbates adverse ventricular remodeling, supporting a cardioprotective role for resident macrophages during cardiac injury. Importantly, resident cardiac macrophages are themselves heterogeneous and contain functionally specialized states. For example, single-cell transcriptomic analysis of fate-mapped resident macrophages identified an insulin-like growth factor 1 (IGF-1)-expressing macrophage state that was selectively activated during hypertensive cardiac stress by angiotensin II and promoted adaptive cardiomyocyte growth through macrophage-derived IGF-1 [33]. Consistent with this specialized function, resident macrophages establish focal interactions with cardiomyocytes and sense mechanical stress through transient receptor potential vanilloid 4 (TRPV4), thereby upregulating IGF-1 expression in CCR2^-^macrophages and contributing to adaptive cardiac remodeling in response to pressure overload [34]. These findings highlight that resident cardiac macrophages are not a homogeneous population but comprise functionally specialized states that respond to local cardiac stress and regulate tissue remodeling through interactions with neighboring cells.

In response to myocardial infarction and other forms of cardiac stress, circulating monocytes are recruited to the injured myocardium and differentiate into CCR2^+^macrophages [35]. CCR2^+^monocyte-derived macrophages constitute a major component of the inflammatory response and contribute to cytokine production, extracellular matrix turnover, and leukocyte recruitment [7]. Inhibition of monocyte recruitment to the injured hearts reduces inflammation and enhances angiogenesis, accompanied by preserved CCR2^-^resident macrophages [36]. Recent studies have further demonstrated that disease-associated monocytes/macrophages promote cardiac fibrosis through macrophage–fibroblast communication. Using single-cell, multiomic, and computational approaches, Amrute et al. demonstrated that CCR2^+^monocyte/macrophage-derived IL-1β drives the activation of fibroblasts expressing fibroblast activation protein (FAP) and periostin (POSTN), thereby contributing to cardiac fibrosis and dysfunction [37]. Similarly, an independent multiomic study identified IL-1β-producing Cx3cr1^+^macrophages as key regulators of fibroblast activation in heart failure and demonstrated that chromatin remodeling within macrophages contributes to the establishment of this profibrotic macrophage–fibroblast communication [38]. Although initially regarded primarily as inflammatory effector cells, subsequent studies demonstrated considerable functional diversity within this population, with distinct subsets contributing to both tissue injury and repair depending on disease stage and local environmental cues. These observations indicate that CCR2^+^monocyte-derived macrophages should not be considered as a uniform inflammatory population but rather as dynamic states shaped by disease stage and local environmental cues.

Recent single-cell analyses have further identified disease-associated macrophage states that emerge during cardiac remodeling. At mid time points after cardiac injury, secreted phosphoprotein 1 (Spp1, osteopontin)^+^monocyte/macrophages and Triggering Receptor Expressed on Myeloid Cells 2 (Trem2)^high^macrophages dominate the lesion, whereas resident-like Cx3cr1^high^macrophages progressively re-establish the homeostatic macrophage compartment during later stages of remodeling [30], [32], [39], [40]. TREM2-expressing macrophages have attracted particular interest because they represent a conserved disease-associated macrophage state implicated in efferocytosis, lipid metabolism, and tissue remodeling across multiple organs [41], [42], [43], [44]. Consistent with these observations, experimental studies demonstrated that myeloid-specific deletion of TREM2 aggravated cardiac dysfunction after MI, whereas TREM2 overexpression improved cardiac remodeling [45]. In this context, TREM2^+^macrophages may influence cardiac remodeling through paracrine signaling. For example, TREM2^+^macrophage-derived itaconate inhibited cardiomyocyte apoptosis and promoted fibroblast proliferation, suggesting coordinated regulation of both cardiomyocyte survival and fibroblast responses. Soluble TREM2 was reported to exhibit cardioprotective effects after myocardial infarction, suggesting that TREM2 may represent a potential therapeutic target. [46]. SPP1^+^macrophages have been implicated in macrophage-fibroblast communication and extracellular matrix remodeling, suggesting a direct contribution to fibrotic progression following cardiac injury [47], [48]. Notably, platelet-derived CXCL4 has been identified as a critical upstream regulator of SPP1^+^macrophage differentiation in the injured heart. Consistent with this concept, ligand-receptor interaction analysis in ischemic kidney injury predicted extensive communication between SPP1^+^macrophages and fibroblasts through SPP1-, FN1-, and SEMA3-associated signaling pathways, supporting a role for these macrophages in coordinating profibrotic multicellular responses [47]. SPP1 is frequently co-expressed with TREM2 in disease-associated macrophage states, and macrophages exhibiting these features have been described as scar-associated macrophages (SAMs) or lipid-associated macrophages (LAMs). Although TREM2- and SPP1-associated macrophage states have emerged as promising regulators of tissue remodeling, their biological functions appear to vary according to the stage of injury and tissue microenvironment. A more comprehensive understanding of these context-dependent properties will therefore be necessary to evaluate their potential as therapeutic targets.

Another population identified by single-cell approaches is the interferon-responsive macrophage state, characterized by increased expression of interferon (IFN)-stimulated genes, including Isg15, Ifit family members, and Irf7, which collectively reflect activation of the type I IFN signaling cascade [39], [49]. The type I IFN response is an innate immune pathway known for its roles in the antiviral response and its association with auto-inflammatory diseases. These cells emerge following myocardial injury and represent a distinct transcriptional program within the monocyte/macrophage compartment. Experimental studies further suggest that activation of the type I IFN pathway contributes to adverse cardiac remodeling after myocardial infarction, highlighting the potential pathological relevance of this macrophage state [50]. On the other hand, IFN-responsive macrophages are thought to communicate with IFNAR-expressing cells through type I IFN signaling. However, the identity of the target cell populations that interact with these macrophages during cardiac remodeling remains largely undefined. Future studies addressing their interactions with cardiomyocytes, fibroblasts, and other immune cells may provide new insights into the mechanisms underlying cardiac remodeling.

Collectively, these findings illustrate how computational approaches have advanced our understanding of disease-associated macrophage states beyond cell identity, providing insight into their context-dependent functions and potential interactions with neighboring cardiac cells.

### Disease-associated neutrophil states and their functional roles in cardiac remodeling

While neutrophils have traditionally been viewed as a relatively homogeneous population of short-lived inflammatory effector cells, recent studies have revealed substantial phenotypic and functional heterogeneity within the neutrophil compartment. Emerging evidence indicates that neutrophils adopt distinct disease-associated transcriptional states during cardiac injury and remodeling, suggesting functions that extend beyond the initiation of acute inflammation.

Single-cell transcriptomic analyses have identified multiple neutrophil states in the infarcted heart, including populations characterized by inflammatory, IFN-responsive, and reparative gene signatures [49], [51]. Notably, transcriptional programs associated with IFN signaling were not restricted to macrophages; a distinct population of interferon-stimulated genes (ISGs)-expressing neutrophils was also identified in infarcted hearts. Although the precise role of these cells in cardiac remodeling remains unclear, their transcriptional profile suggests responsiveness to IFN signaling and highlights additional layers of neutrophil heterogeneity within the injured myocardium. Studies in other disease settings have demonstrated that IFN signaling profoundly regulates neutrophil survival and ROS production [52], [53], raising the possibility that ISG-expressing neutrophils participate in immune regulation through interactions with neighboring cells rather than acting solely as inflammatory effector cells. Whether these cells influence neighboring cardiomyocytes, fibroblasts, endothelial cells, or other immune populations through IFN-dependent signaling during cardiac remodeling remains to be determined. These findings challenge the traditional view of neutrophils as terminally differentiated cells with limited functional diversity and instead support the existence of context-dependent neutrophil states that evolve throughout the remodeling process.

Among these populations, Siglec-F-expressing neutrophils have attracted particular attention because of their unique transcriptional profile and potential role in tissue repair. Although Siglec-F, a murine counterpart of human SIGLEC8, is traditionally recognized as an eosinophil marker, recent studies have identified Siglec-F-expressing neutrophils in several pathological conditions, including lung cancer [54], [55]. Using single-cell transcriptomic analysis, Vafadarnejad and colleagues identified a distinct Siglec-F^high^neutrophil population that emerged during the later stages of myocardial infarction and exhibited a higher phagocytic and ROS-production capacity [51]. Recent studies suggest that Siglec-F expression is acquired locally within tissues and may be induced by factors such as TGF-β and granulocyte-macrophage colony-stimulating factor (GM-CSF) [56], [57], indicating that this disease-associated neutrophil state is shaped by the local tissue microenvironment. Thus, Siglec-F^high^neutrophils may represent a tissue-adapted neutrophil state whose functional properties are determined by disease-specific microenvironmental cues. Emerging evidence suggests that the biological functions of Siglec-F-expressing neutrophils are highly context-dependent. In the heart, these cells have been implicated in periodontitis-aggravated post-infarction remodeling through TNF-α-mediated fibroblast activation and collagen production [21]. Conversely, Siglec-F-expressing neutrophils facilitate the resolution of acute lung injury through macrophage colony-stimulating factor (M-CSF)-mediated monocyte-to-macrophage differentiation and lung epithelial restitution [57], whereas they contribute to fibrotic progression in the kidney [56]. These observations suggest that the biological activities of Siglec-F-expressing neutrophils are determined not only by their intrinsic transcriptional programs but also by context-dependent interactions with neighboring fibroblasts, epithelial cells, macrophages, and other immune populations. Interestingly, disease-associated neutrophil states are themselves subject to regulation by other immune cells. For example, G protein-coupled receptor 43 (GPR43)-expressing eosinophils have recently been shown to suppress the accumulation of Siglec-F^high^neutrophils [58], indicating that the induction and maintenance of this neutrophil state are regulated by interactions with other immune cells. Together, these findings suggest that the mechanisms regulating the induction, maintenance, and functional specialization of Siglec-F^high^neutrophils remain incompletely understood. Future studies integrating spatial transcriptomics with computational ligand-receptor analyses will be important for identifying potential cellular interactions that regulate these cells and determining how such interactions contribute to their context-dependent roles in tissue remodeling.

Collectively, these findings indicate that neutrophils, similar to macrophages, undergo dynamic state transitions during cardiac remodeling and that their functions are shaped by context-dependent interactions with neighboring cell populations within the injured myocardium.

### GARP-expressing myeloid (GEM) cells: discovery, biological functions, and translational relevance

The identification of diverse disease-associated myeloid states has shifted the focus of cardiac immunology from individual myeloid populations toward understanding the mechanisms underlying their emergence and contributions to tissue remodeling. Despite the growing appreciation of myeloid cell heterogeneity, therapeutic strategies targeting inflammation have provided only limited clinical benefit in patients with cardiovascular disease. These observations suggest that functionally important myeloid populations involved in cardiac remodeling remain incompletely characterized.

To further explore previously unrecognized myeloid cell states, our group performed single-cell RNA sequencing focusing exclusively on cardiac myeloid cells during pathological remodeling [59]. This analysis identified a distinct myeloid population characterized by high expression of GARP, which we termed GARP-expressing myeloid (GEM) cells. Similar GEM cells were subsequently identified in independent public single-cell datasets [60], supporting the existence of this previously unrecognized disease-associated myeloid state. GEM cells emerged in the subacute phase after MI and exhibited a transcriptional profile that was clearly distinguishable from established macrophage and neutrophil states, suggesting that they represent a previously unrecognized myeloid population with unique biological functions. Transcriptomic analysis demonstrated that GEM cells highly expressed fibroblast-associated genes, including Col1a1, S100a4 (FSP1), and Pdgfra, while remaining within the CD11b^+^myeloid compartment. This hybrid molecular signature suggested similarities to fibrocytes, which exhibit both hematopoietic and mesenchymal characteristics.

Fibrocytes were originally described as circulating bone marrow-derived cells that co-express hematopoietic markers together with extracellular matrix proteins such as collagen 1 [61], [62]. Since their initial description, fibrocytes have been implicated in tissue repair, fibrosis, angiogenesis, and immune regulation in multiple organs. In the kidney, fibrocytes have been shown to accumulate in fibrotic lesions and contribute to renal fibrosis through CCL21/CCR7-dependent recruitment [63], [64]. In patients with cardiovascular diseases, including hypertension and coronary heart disease, circulating fibrocytes have also been reported to be increased, although their functional roles in cardiac remodeling remain poorly understood [65], [66]. Despite these findings, the identity of fibrocytes remains controversial because of considerable phenotypic overlap with monocytes and macrophages, and the lack of universally accepted lineage-specific markers [67]. Computational integration with publicly available single-cell datasets [68] suggested a close developmental relationship between putative fibrocytes and GEM cells, with pseudotime analysis supporting a potential transition toward a GARP-expressing state during cardiac remodeling. However, whether GEM cells represent activated fibrocytes or a distinct fibrocyte-lineage myeloid population remains to be determined.

GARP is a cell surface docking receptor that binds latent TGF-βs and facilitates their activation. GARP is expressed by regulatory T cells (Tregs) [69], [70], activated B cells [71], platelets, endothelial cells [72], and several types of tumor cells [73]. In Tregs, GARP presents latent TGF-β on the cell surface and promotes localized TGF-β activation, thereby modulating the behavior of neighboring cells and contributing to immunosuppressive activity. Systemic GARP-deficient mice die shortly after birth because of cleft palate resulting from impaired apoptosis during palatogenesis [74], highlighting an essential role for GARP in tissue morphogenesis in addition to immune regulation. These observations prompted us to investigate whether GARP-expressing myeloid cells represent a functionally distinct population involved in cardiac remodeling.

To investigate the functional significance of GEM cells, we generated myeloid-specific GARP-deficient mice. Although GARP deficiency did not affect cardiac development or basal cardiac function, it markedly attenuated post-infarction remodeling, leading to reduced fibrosis, improved cardiac function, and enhanced survival. These findings identified GEM cells as a pathogenic myeloid population that promotes adverse cardiac remodeling. Mechanistically, single-cell transcriptomic analysis demonstrated that GARP deficiency did not substantially alter the transcriptional identity of GEM cells but instead reduced neutrophil accumulation within the infarcted myocardium. Rather than directly promoting neutrophil recruitment through TGF-β-related ligand-receptor interactions, GEM cells activated TGF-β/Smad signaling in cardiomyocytes, leading to CXCL1 induction and subsequent neutrophil recruitment. Ligand-receptor interaction analysis revealed no substantial changes in the interactions between GEM cells and monocytes or macrophages following GARP deficiency. In addition to regulating neutrophil recruitment, GEM cells directly promoted cardiomyocyte apoptosis through a TGF-β-dependent mechanism. Together, these findings support a model in which GEM cells contribute to pathological remodeling by simultaneously enhancing cardiomyocyte injury and neutrophil-mediated inflammation through intercellular communication. Notably, GARP-mediated apoptosis appeared to be largely restricted to cardiomyocytes rather than non-cardiomyocytes, including fibroblasts. Although the mechanism underlying this cell type specificity remains unclear, differences in the dynamics of TGF-β/Smad signaling between quiescent and proliferating cells [75] may influence cellular susceptibility to GARP-mediated TGF-β activation. Further studies are needed to clarify the molecular mechanisms underlying the interactions between GEM cells and other cardiac cell populations. Importantly, although accumulating evidence suggests that fibrocytes participate in inflammatory responses by regulating the production of inflammatory mediators, such as IL-6 and TNF-α [76], [77], direct regulation of parenchymal cell survival has not been recognized as a canonical fibrocyte function. In contrast, GEM cells promoted cardiomyocyte apoptosis through GARP-dependent TGF-β signaling, suggesting that they possess biological functions distinct from those of conventional fibrocytes despite their shared transcriptional features.

To investigate the clinical relevance of GEM cells, we analyzed publicly available human transcriptomic datasets. Spatial transcriptomic analysis of human infarcted myocardium demonstrated regions in which LRRC32 (GARP) and ITGAM (CD11b) were co-expressed, supporting the presence of GARP-expressing myeloid cells in human myocardial infarction. Furthermore, analysis of single-cell RNA-seq data from patients with acute kidney injury identified a distinct GARP-expressing myeloid population that shared transcriptional and signaling characteristics with murine cardiac GEM cells. These findings suggest that GEM cells are not unique to the infarcted heart but may represent a conserved injury-associated myeloid state that emerges across multiple organs, raising the possibility that GARP-expressing myeloid programs are conserved features of tissue injury and remodeling.

Recent technological advances, including single-cell transcriptomic and spatial analyses, have revealed remarkable heterogeneity among cardiac myeloid cells and identified multiple disease-associated myeloid states that emerge during cardiac remodeling. These populations exhibit distinct molecular programs and may contribute to tissue remodeling through potential interactions with cardiomyocytes, endothelial cells, fibroblasts, and other immune cells. Among these disease-associated populations, GARP-expressing myeloid (GEM) cells represent a newly identified myeloid state characterized by a fibrocyte-like transcriptional signature. Mechanistically, GEM cells contribute to adverse cardiac remodeling by promoting cardiomyocyte apoptosis and neutrophil recruitment through GARP-dependent activation of TGF-β signaling. This schematic summarizes the current understanding of disease-associated myeloid states and highlights GEM cells as an example of a previously unrecognized myeloid state that contributes to pathological cardiac remodeling through specialized intercellular communication. The figure was created with BioRender.com.

## Future perspectives

Recent advances in single-cell and spatial transcriptomic technologies have transformed our understanding of cardiac myeloid biology by revealing numerous disease-associated myeloid states with distinct molecular and functional characteristics. Although the biological functions of several disease-associated myeloid populations and their interactions with neighboring cells are beginning to be elucidated, these findings have largely emerged from studies focusing on individual cell populations and their associated signaling pathways. Consequently, how multiple myeloid populations cooperate within integrated multicellular signaling networks to coordinate cardiac remodeling remains poorly understood. Moving beyond the identification of additional specialized myeloid populations to define these intercellular circuits will therefore represent a major challenge for the next generation of cardiovascular research.

Despite the rapid expansion of transcriptomic atlases, our understanding of intercellular communication remains incomplete. Single-cell RNA sequencing provides high-resolution information regarding cellular identity and activation states but cannot directly determine which cellular interactions actually occur within the tissue microenvironment. Consequently, many proposed communication pathways remain largely inferred from transcriptional data. As spatial transcriptomic, multiplex imaging, photo-isolation chemistry (PIC)-based spatial transcriptomic approaches [78], and emerging single-cell and spatial proteomic technologies [79], [80], [81] continue to mature and become more widely accessible, their integration will provide unprecedented opportunities to define the spatial organization of disease-associated cell states and elucidate the molecular interactions that drive cardiac remodeling. Furthermore, the development of accessible computational frameworks and analytical pipelines will be essential to extend these approaches beyond specialized bioinformatics groups. Lowering the barriers to computational analysis will enable a broader community of experimental researchers to incorporate transcriptomic and spatial datasets into their own biological investigations, facilitating iterative cycles of computational discovery and experimental validation and accelerating our understanding of the multicellular mechanisms underlying cardiac remodeling.

Among the disease-associated myeloid populations identified to date, GEM cells provide an illustrative example of how previously unrecognized cellular states may regulate cardiac remodeling through specialized interactions with neighboring cells within the injured myocardium. Unlike conventional macrophage or neutrophil states, GEM cells exhibit a hybrid molecular signature with similarities to fibrocytes while promoting cardiomyocyte apoptosis and neutrophil recruitment through GARP-dependent TGF-β signaling. These findings suggest that specialized myeloid states may acquire unique biological functions by establishing distinct patterns of intercellular communication with neighboring cells within the injured myocardium. Nevertheless, many fundamental questions remain unanswered. The developmental origin of GEM cells has not been fully established, and it remains unclear whether they represent activated fibrocytes, a distinct fibrocyte-lineage myeloid population, or a transient injury-induced state. Likewise, the molecular mechanisms governing GARP induction, latent TGF-β activation, and the downstream signaling pathways responsible for GEM cell-mediated communication remain to be elucidated. Because GARP expression in Tregs is tightly regulated by activation-dependent pathways [82], [83], determining whether analogous mechanisms operate in GEM cells may provide important insights into the establishment and functional specialization of this unique myeloid population. Addressing these questions will not only clarify the biology of GEM cells but also advance our broader understanding of how specialized myeloid cell states coordinate pathological cardiac remodeling.

Another important challenge is to determine the extent to which injury-associated myeloid states are conserved across organs and pathological conditions. Although transcriptomic similarities between cardiac and renal GEM cells suggest the existence of a conserved injury-associated myeloid program, it remains unknown whether comparable populations participate in other pathological conditions, including pressure overload-induced heart failure, cardiomyopathies, chronic kidney disease, lung injury, or systemic inflammatory diseases. Comparative analyses across tissues and disease models may therefore reveal conserved cellular programs and interaction mechanisms that underlie pathological remodeling beyond the cardiovascular system.

Finally, the identification of disease-associated myeloid states raises important therapeutic opportunities. Previous attempts to broadly suppress inflammation in cardiovascular disease have generally yielded limited clinical benefit, partly because inflammatory cells perform both protective and detrimental functions during tissue repair. A more precise understanding of myeloid cell heterogeneity may enable selective modulation of pathogenic cellular states while preserving beneficial immune responses. Rather than targeting myeloid cells indiscriminately, future therapeutic strategies may focus on selectively modulating pathological cellular interaction networks that connect myeloid cells with cardiomyocytes, fibroblasts, endothelial cells, and other immune populations.

Collectively, recent advances have established that cardiac remodeling is an emergent property of coordinated multicellular interactions rather than the consequence of dysfunction within individual cell types. Defining how disease-associated myeloid states are generated and function within multicellular tissue environments will provide a foundation for the development of next-generation therapeutic strategies.

## CRediT authorship contribution statement

**Masanori Obana:** Writing – review & editing, Writing – original draft, Funding acquisition, Conceptualization. **Masashi Tomimatsu:** Writing – review & editing. **Shota Tanaka:** Writing – review & editing. **Keisuke Shirakura:** Writing – review & editing. **Yoshiaki Okada:** Writing – review & editing. **Yasushi Fujio:** Writing – review & editing, Funding acquisition, Conceptualization.

## Author contributions

M.O. and Y.F. drafted the manuscript; all authors edited and revised the manuscript; all authors approved the final version of the manuscript.

## Declaration of Generative AI and AI-assisted technologies in the writing process

During the preparation of this work, the authors used ChatGPT for English language editing and assistance with literature searches. The authors reviewed and edited the output as needed and take full responsibility for the content of the published article.

## Funding

M.O. receives funding from the 10.13039/501100001691JSPS/KAKENHI (26K09627), and Y.F. receives funding from the JSPS/KAKENHI (25K02446).

## Declaration of Competing Interest

The authors have no conflicts of interest.
